# Effect of a 12-Week Online Walking Intervention on Health and Quality of Life in Cancer Survivors: A Quasi-Randomized Controlled Trial

**DOI:** 10.3390/ijerph15102081

**Published:** 2018-09-21

**Authors:** Lauren J. Frensham, Gaynor Parfitt, James Dollman

**Affiliations:** 1 School of Psychology Social Work and Social Policy, University of South Australia, Adelaide, SA 5001, Australia; 2 Sansom Institute for Health Research, School of Health Sciences, University of South Australia, Adelaide, SA 5001, Australia; gaynor.parfitt@unisa.edu.au (G.P.); james.dollman@unisa.edu.au (J.D.)

**Keywords:** cancer, survivor, walking, physical activity, pedometer, intervention

## Abstract

Cancer survivors are at an increased risk of experiencing physical and psychological ill-effects following cancer treatment. Rural cancer survivors are at a greater risk of future health problems following a cancer diagnosis compared to their urban counterparts. Physical activity has been targeted as a health promotion priority in cancer survivors. Research indicates that a large portion of cancer survivors do not meet physical activity recommendations. The purpose of this quasi-randomized controlled trial was to test the effectiveness of an online 12-week walking intervention designed for cancer survivors, and to explore its impact on physical health indicators and quality of life outcomes. Steps Toward Improving Diet and Exercise among cancer survivors (STRIDE) is an online resource designed according to Social Cognitive Theory and Self Determination Theory, based on individualized step goal setting. Measures of physiology, physical fitness, and quality of life were taken at the baseline, post-intervention, and three-month follow-up in an Intervention group (*n* = 46) and active Control group (*n* = 45). The Control group was provided with a pedometer but did not have access to the online program. Three-factor repeated measures ANOVAs indicated that there were improvements in physical fitness (*p* < 0.01), systolic blood pressure (*p* < 0.01), diastolic blood pressure (*p* < 0.01), waist girth (*p* < 0.01), mental health (*p* < 0.05), social functioning (*p* < 0.01), and general health (*p* < 0.01), but an increase in bodily pain (*p* < 0.01), from the baseline to week 12 and the three-month follow-up, irrespective of group allocation. Pedometer interventions, delivered with or without online support and step goal setting, show promise for improving the overall health of cancer survivors, at least in the short term.

## 1. Introduction

Approximately 40% of the general population will receive a cancer diagnosis [1]. Advances in cancer detection, treatment, and management have increased the five-year relative survival rate to approximately 70% [2,3]. As the number of cancer survivors continues to rise, it is crucial to explore methods of improving their health and psychological wellbeing throughout survivorship.

Cancer survivors often experience adverse side effects including pain [4] and fatigue [5], as well as an increased risk of functional impairment, morbidity, and premature mortality [6]. Many cancer survivors experience psychological distress and impaired social functioning [7], which can negatively impact their quality of life [8]. Reviews of the literature show that physical activity can reduce such physical limitations, obesity, secondary cancers, and premature mortality in cancer survivors [9,10]. Previous studies also indicate that physical activity can improve quality of life (QOL) across different domains of functioning (e.g., physical, emotional, psychological, social, and spiritual) [11]. Given these health benefits, cancer organizations worldwide have provided recommendations and guidelines for cancer survivors to incorporate physical activity into their daily lives. However, it is estimated that only 10–20% of cancer survivors will be active after treatment [12,13]. Cancer survivors living in rural and remote areas may be at an even higher risk of being insufficiently active and experiencing negative long-term effects of cancer treatment compared to their urban counterparts [14,15]. This disparity in health outcomes between metropolitan and rural cancer survivors may be attributable to geographical isolation, limited access to services and facilities, and the burden of travel distances [16,17]. Given the known benefits of physical activity on health outcomes in cancer survivors and the inequality in health outcomes between metropolitan and rural cancer survivors, there is a need for accessible health promotion interventions that meet the specific needs of both metropolitan and rural cancer survivors.

This quasi-randomized controlled trial aimed to investigate the effectiveness of an online tool, Steps Toward Improving Diet and Exercise (STRIDE), designed to improve physical activity (primarily walking) among cancer survivors. It was hypothesized that the STRIDE intervention would lead to an improvement in physical health indicators (blood pressure, body composition), QOL outcomes (physical, psychosocial, social), and physical fitness from the baseline to the end of intervention and at the three-month follow-up.

## 2. Materials and Methods

### 2.1. Participants and Setting

Participants were recruited via flyers, cancer support groups, newspaper advertisements, radio presentations, and allied health personnel in metropolitan and rural regions of South Australia. Participants were telephone screened for eligibility and were required to: (a) have had a cancer diagnosis (excluding skin cancer); (b) not be currently receiving active treatment such as surgery, chemotherapy, or radiotherapy; (c) be insufficiently active, defined as engaging in less than 20 sessions of exercise over the past month (one session is 30 min duration) [18]; (d) not be pregnant or planning to become pregnant during the study; (e) have no physical/psychiatric conditions impairing their ability to participate; (f) not have any known contraindications for exercise as assessed by Stage 1 of the Adult Pre-Exercise Screening Tool [19]; (g) provide informed consent; (h) have access to a computer with the Internet; and (i) be able to obtain medical clearance.

**Figure 1:**
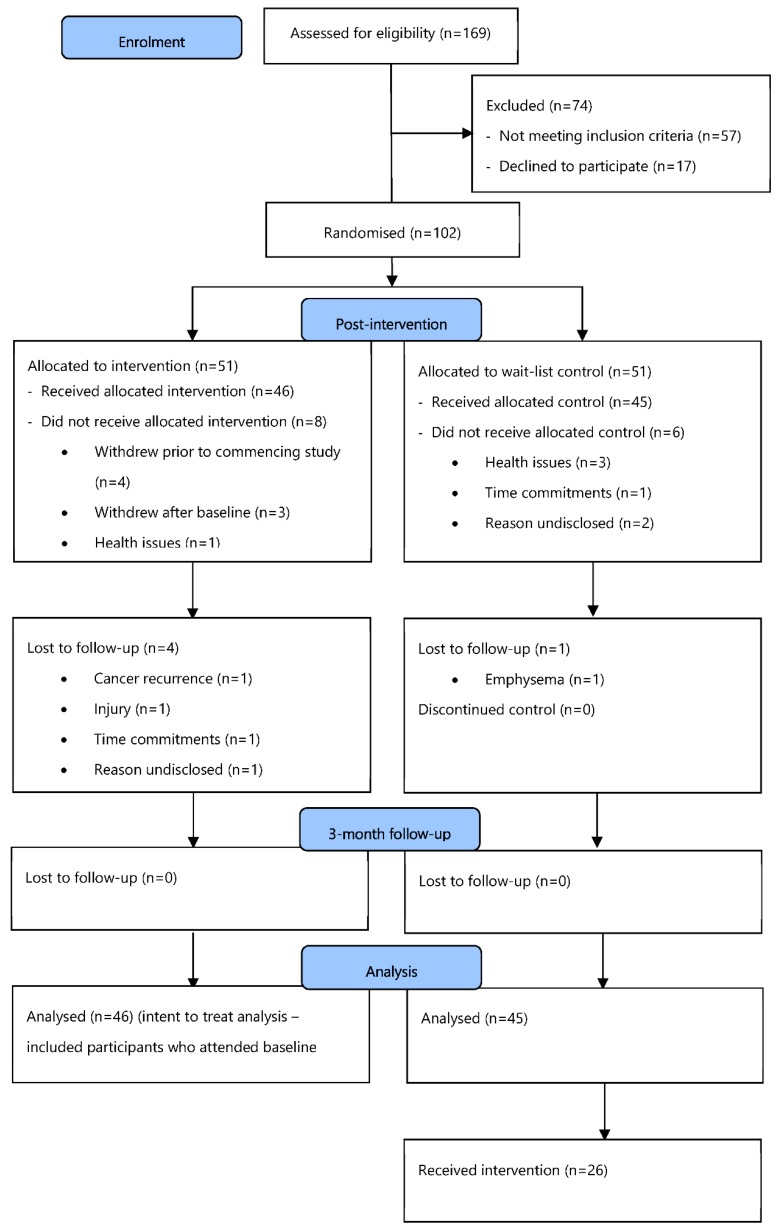
Consort diagram: Participant flow chart.

The STRIDE study was a quasi-randomized controlled intervention trial. Details of the protocol have been previously published [20]. Cancer survivors willing to participate were allocated to either the intervention group or a wait-list control group. Intervention participants received a pedometer and access to an online resource that included an interactive physical activity diary to facilitate individualized goal setting, self-monitoring/management, and reinforcement for behavior change. Participants were advised to enter their daily step counts, as well as their perception of exertion using Borg’s ‘6–20 Rating of Perceived Exertion (RPE) Scale’ [20,21]. These inputs (daily steps, RPE during walking, and daily affect) were used to generate individually tailored target steps/day for the following week, which maintained exertion between RPE 11 (light) and 13 (somewhat hard) on the RPE scale; the “bandwidth” within which people have the most positive response to exercise [22,23,24]. Control participants were able to use their pedometer but did not have access to the online STRIDE program with goal setting. In total, 91 participants were recruited and randomized to groups. The flow of participants through the various recruitment, screening, and group allocation stages is presented in Figure 1.

Eligible volunteers were provided with further study information, a consent form, and pre-exercise screening form, and were asked to obtain medical clearance from their treating doctor using a standardized medical clearance form. Pre-exercise screening was conducted using Stage 1 of the Sports Medicine Australia Pre-Exercise Screening System [19]. The first stage of the screening system was designed to screen out those people who were at a high risk of exercise-related complications due to underlying cardiovascular, cerebrovascular, respiratory, or metabolic diseases, and also pre-existing injuries. Both exercise screening and medical clearance were requested as the sample was considered to be high risk.

### 2.2. Randomization and Blinding

A block design with an allocation weight of 3:3 was used to generate treatment allocation. According to the PEDro scale [21], this study was considered quasi-experimental as it was not possible for key researchers to be blinded to participants’ group allocation because they were performing the information sessions relevant to each group. Nor was it possible to blind participants to group allocation, particularly in rural locations, where participants were likely to know each other and communicate regularly. However, six research assistants performing physical and questionnaire assessments were blinded to group allocation.

### 2.3. Ethical Approval

This study received ethical approval from the University of South Australia Human Research Ethics Committee prior to study commencement (Ref. 0000031039) and was registered with the Australian New Zealand Clinical Trials Registry (Trial Registration: ACTRN12613000473763).

### 2.4. Protocol

All participants (intervention and control groups) attended two baseline workshops, one week apart. Each workshop lasted approximately 90 min. At the first workshop, health measures were taken (blood pressure, weight, height, waist and hip circumference) and fitness was assessed using the six-min walk test (6MWT) [22]. Participants completed questionnaires about their physical functioning and Quality of Life (QOL). They were provided with a sealed pedometer (New-Lifestyles NL-1000 pedometer, New Lifestyles Inc., Lees Summit, MO, USA), which they returned at workshop two with a pedometer log sheet. The advantages of the NL-1000 pedometer are that: it stores the previous seven days of data and therefore does not require the participant to record steps each day, a process that adds to participant burden; and it requires a complex sequence of button presses to alter internal settings, ensuring a low risk of accidental or deliberate tampering and therefore a low risk of data loss or contamination. The NL series pedometer has extensive empirical backing for validity and inter-instrument reliability, and displays the accuracy required to detect changes in step counts that are typical of walking interventions [23].

At workshop two, all participants (intervention and control groups) were provided with lifestyle information and a pedometer (Yamax Digiwalker SW700). Only those in the intervention group were instructed on using the STRIDE website, including how to log their steps and other relevant data. On the basis of this information, they were emailed daily step goals that they were encouraged to achieve. Following the 12 weeks of the intervention period, all measures were repeated, and again after six months (i.e., three-months post-intervention). At the conclusion of six months, the control group was offered the STRIDE program.

### 2.5. Intervention

#### 2.5.1. STRIDE Website

The STRIDE program comprises two main components, the STRIDE website and weekly step goals. Participants used a pedometer to monitor the number of steps taken each day and recorded this information on the step log on the STRIDE website. Participants reported their Rating of Perceived Exertion (RPE) during walking and their daily affect. The step log included a graph of average weekly steps as feedback on progress during the intervention. An online forum provided an opportunity for participants to share experiences and offer peer support. A virtual noticeboard allowed community centers, health providers, and walking groups to advertise activities and events. The website included healthy eating information through the Cancer Council Australia’s nutrition guidelines, based on recommendations in The Australian Guide to Healthy Eating [24].

#### 2.5.2. Step Goals

Personalized step targets were created using individuals’ RPE and affect in combination with logged daily steps. RPE was determined using Borg’s 6–20 Ratings of Perceived Exertion scale [25,26] that numerically quantifies the effort, strain, discomfort, and/or fatigue experienced during physical activity to represent how difficult or easy the activity is perceived to be. RPE has been used to create target exercise intensities in a range of populations including cardiac patients and patients with chronic obstructive pulmonary disease [26,27]. Research has indicated that affective state (feeling good/bad) influences exercise behavior and motivation [28]. Given that cancer survivors may experience wide variability in affective state, how the individual is feeling was considered when setting individualized step goals. Participants rated their affective state each day on a scale of +5 (‘Very good’) to −5 (‘Very bad’) [29]. Logged data (daily steps, RPE during walking and daily affect) were used to generate individual target steps/day for the following week that maintained exertion at between RPE 11 (light) and 13 (somewhat hard), a bandwidth within which people typically have a positive affective response to exercise [30]. Three-tiered step goals were provided using the affect scale; a goal for when the participant was feeling ‘bad’ (i.e., minus on the affect scale), ‘neutral’, and ‘good’. This method was employed to ensure that the goals were perceived to be challenging yet achievable, by acknowledging variability in affective state from one day to the next. If participants did not meet the step goals for the previous week on the majority of logged days, the goals for the following week did not differ from the previous week or may have been lowered. The first week served as baseline data from which the first goals were set, with 0%, 5%, and 10% used as a guide for progression across the 12 weeks of the intervention on ‘bad’, neutral’, and ‘good’ days, respectively.

### 2.6. Outcome Measures

Outcome measures were assessed at three time points: baseline, week 12 (end of intervention), and at a three-month follow-up.

#### 2.6.1. Physical Activity

Participants wore a sealed pedometer for seven consecutive days (five week days and two weekend days). Minimum wear time, as recorded by a log sheet, was defined as 10 h per day (based on previous literature) [31] for four of the seven days, one of which must have been a weekend day. The results of intervention effects on steps/day in this study have been reported elsewhere [32].

#### 2.6.2. Anthropometry

The following anthropometric measures were taken: standing stretch stature using a portable stadiometer (SECA, Hamburg, Germany); body weight (Tanita UM-108, Tanita Corporation, Tokyo, Japan); and waist and hip girths (Executive Thinline 2 m W606pm, Lufkin Tape, Apex, NC, USA). All physical measurements were taken according to the International Society for the Advancement of Kinanthropometry [33]. All anthropometric measures were taken in privacy. A minimum of two measures was taken at each site. A third measure was taken if the difference between the first and second measures was greater than 0.5 cm for stature, waist, and hip girths.

#### 2.6.3. Physiological Measures

Resting blood pressure was measured using automated sphygmomanometers (Dinamap Pro 100, GE Medical Systems Information Technologies and Critkon Company, Tampa, FL, USA) after participants had been seated for five minutes. Unless contraindicated, blood pressure was taken on the left arm, with the middle of the cuff on the upper arm at the level of the right atrium. A range of cuff sizes was available to suit participants’ arm circumference. Measures were repeated until there were two readings within 5 mmHg for both systolic and diastolic pressure, up to a maximum of four times.

#### 2.6.4. Physical Fitness

The 6MWT is a valid, responsive, interpretable self-paced test that quantifies functional exercise capacity as the distance walked in six minutes [34]. The test was performed over a 20 m, level, straight course within an enclosed corridor adapted from the protocol described by the American Thoracic Society [22]. During each 6MWT, participants received standardized instructions and encouragement. At the end of the test, participants were asked to rate their perceived exertion on Borg’s scale [25].

#### 2.6.5. Functional Status and Quality of Life (QOL)

Functional status and QOL were measured using the Australian adaptation of the ‘Short Form (36) Health Survey’ (SF-36v2) [35,36]. The SF-36v2 is a brief, broad, generic measure of health status, comprising 36 items that represent eight domains of health and quality of life: physical functioning; role limitations because of physical health problems; bodily pain; social functioning; general mental health (psychological distress, psychological wellbeing); role limitations because of emotional problems; vitality (energy/fatigue); and general health perceptions. By combining these eight domains, two further subscales are derived: Physical Component Scale (PCS) and Mental Component Scale (MCS).

Where necessary, scales were reverse-scored so that a higher score indicates better health (i.e., the lower the score, the more disability and the higher the score, lower disability). The SF-36 has been used in other studies of lifestyle interventions among cancer survivors and in self-management interventions in chronic disease [37]. Consistent with a reliability analysis of the American version, all scales of the Australian version of the SF-36 demonstrate high internal consistency, with Cronbach’s alpha ranging from 0.81 (General Health) to 0.92 (Physical Functioning and Bodily Pain). The median of item-scale correlations within each scale ranged from 0.61 (Mental Health) to 0.82 (Role-Physical) [35].

The SF-36 data were scored using the ‘RAND-36 Health Survey’ scoring key [38]. This involved a two-step process. First, pre-coded numeric values were recoded per the RAND-36 scoring key. Each item was scored on a 0 to 100 range, such that the lowest and highest scores were set at 0 and 100, respectively. Scores represent the percentage of the total possible score. In step 2, items in the same scale were averaged together to create the eight-scale scores. In cases of missing values, the average of the other items for that scale was entered. Thus, scale scores represent the average for all items in the scale that the respondent answered [39].

### 2.7. Demographics

Socio-demographic variables were assessed by a self-report questionnaire: age, ethnicity, location (metropolitan, rural), marital status (married, living with partner, separated, divorced, widowed, or never married), level of education (never attended school, some primary school, completed primary school, some high school, completed high school, trade/certificate or diploma, university degree, or higher university degree), employment status (full-time, part-time, self-employed, home-duties, student, unemployed, retired, or unable to work), and smoking status (daily, occasionally, used to smoke, tried, or never smoked). Details on cancer type, cancer stage at diagnosis, and treatment type(s) were collected at the baseline.

### 2.8. Data Analysis

Data were analyzed using SPSS (version 21, IBM Corporation, Chicago, IL, USA). The per-protocol analysis was applied and the significance level was set at 0.05. Baseline comparisons of treatment groups based on demographic characteristics were performed using independent *t*-tests. Assumptions of sphericity in an analysis of variance (ANOVA) with repeated measures were tested using Mauchly’s test, and, if violated, the Greenhouse-Geisser correction was used.

Three-factor (condition: Intervention, Control) by time (baseline, week 12, three-months post-intervention) by region (metropolitan, rural) ANOVA with repeated measures on time was used to examine the hypothesized effect of the intervention on outcome variables: fitness, BMI, waist girth, blood pressure, and QOL. Condition (Intervention, Control) by time repeated measures ANOVA was used to examine differences in these outcomes between the intervention and control groups over time. All models were controlled for marital status, level of education, employment status, and smoking status.

## 3. Results

### 3.1. Participants

The socio-demographic, health, cancer-specific, and comorbidity profiles of the intervention and control participants are presented in Table 1 and Table 2 below. Although the mean age of participants in the intervention and control groups did not differ significantly (65.2 years compared with 66.1 years respectively), the age range was lower in the intervention group (29–78 years) compared with the control group (44–86 years). Tumor characteristics were similar for both intervention and control groups, with a majority of participants being breast cancer survivors (45.7% and 37.8% respectively) and prostate cancer survivors (19.6% and 24.4% respectively). Most participants had been diagnosed with cancer only once (84.8% in the intervention group and 86.7% in the control group). Time since first cancer diagnosis was similar in both groups, as were comorbidity characteristics.

### 3.2. Reliability of Constructs Derived from SF-36v2

Internal reliability of the eight domains from the SF-36v2 in this sample was tested using Cronbach’s α. The reliability was considered to be adequate if the α value was >0.70 [40]. The Cronbach’s α coefficients for seven of the eight dimensions of the SF-36v2 were >0.70, but the social function dimension exhibited a coefficient of 0.61 and was excluded from analyses (Table 3).

### 3.3. Visits to STRIDE Website

On average, participants logged onto the website a total of 53 times over the 12-week program. This equates to an average of 4.4 times per week. Metropolitan participants logged onto the website more frequently (average of five times per week) compared to rural participants (average of three times per week).

### 3.4. Effect of the Intervention on Outcome Variables

The time by region interaction term did not reach significance in any of the models and was consequently removed. There were no significant region main effects.

#### 3.4.1. Health Variables

**Figure 2:**
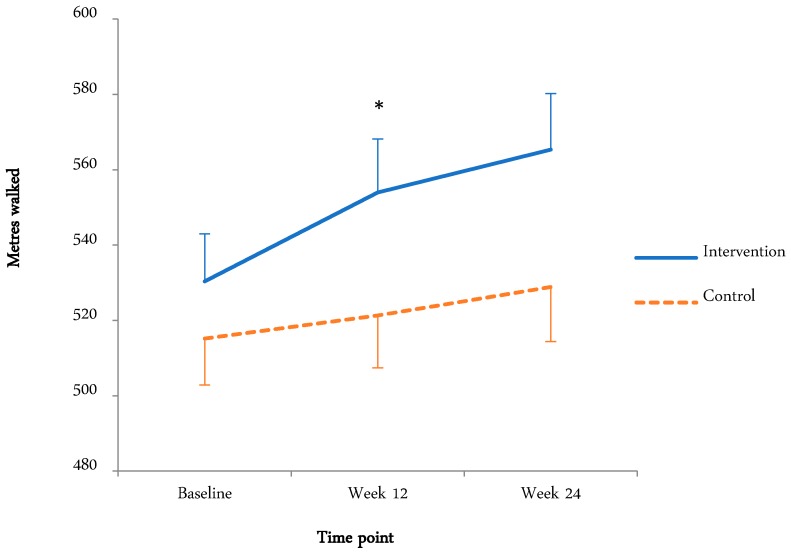
Fitness for intervention and control groups at baseline, week 12, and three-month follow-up. * Indicates significant time by condition interaction.

**Figure 3:**
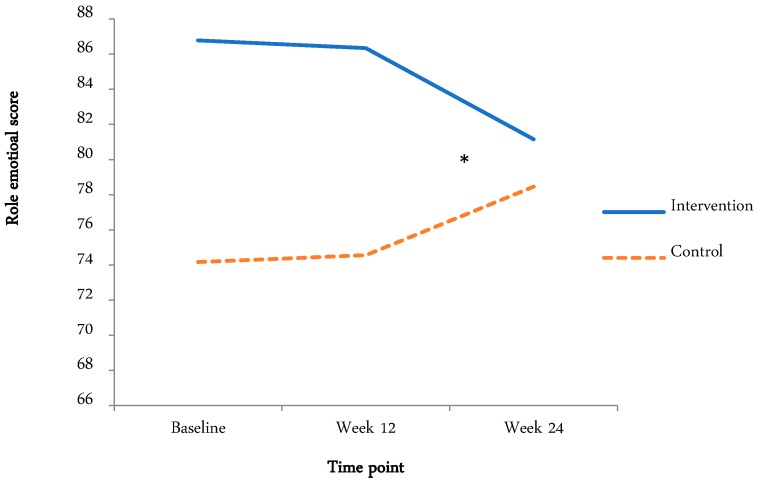
Role emotional score for intervention and control groups at baseline, week 12, and three-month follow-up. * Indicates significant time by condition interaction.

The means and standard deviations and effects (time and condition main effects, time by condition interaction) are tabulated below (Table 4 and Table 5). Where there was a time by condition interaction, graphical representation is displayed (Figure 2 and Figure 3).

#### 3.4.2. Physical Fitness (6MWT)

Figure 2 displays the fitness for both groups at the baseline, week 12, and the three-month follow-up. There was a significant time main effect (F(1.8,138.3) = 12.4, *p* < 0.01, η_p_^2^ = 0.14), with meters walked increasing from the baseline (522.6 ± 78.7 m) to week 12 (537.2 ± 89.7 m) and the three-month follow-up (546.6 ± 94.1 m). The time by condition interaction approached significance (F(1.8, 138.3) = 2.65, *p* = 0.08, η_p_^2^ = 0.33). This appears to be due to the increase in distance walked from the baseline to week 12 in the intervention group.

#### 3.4.3. SF-36 Scores

Table 5 shows the SF-36 scores for intervention and control groups at baseline, week 12, and follow-up. The means and standard deviations and effects (time and condition main effects, time by condition interaction) are indicated.

#### 3.4.4. Physiological Variables

- Blood pressure

As systolic and diastolic blood pressure are likely to be related, a three-way ANOVA was conducted to test if there was a condition effect on systolic and diastolic blood pressure over time. The ANOVA revealed no significant time by condition interaction or condition main effects. An ANOVA with repeated measures on time for systolic blood pressure resulted in a significant time main effect (F(1.8,140.4) = 13.3, *p* < 0.01, η_p_^2^ < 0.14), with systolic blood pressure decreasing from the baseline (139.24 ± 17.9) to week 12 (133.81 ± 18.02) and three months post-intervention (131.21 ± 16.04). An ANOVA with repeated measures on time with diastolic blood pressure resulted in a significant time main effect (F (2,160) = 11.5, *p* < 0.01, η_p_^2^ < 0.13), with diastolic blood pressure decreasing from the baseline (80.53 ± 10.21) to week 12 (78.33 ± 10.85) and three months post-intervention (76.24 ± 10.59).

- BMI

An ANOVA with repeated measures on time resulted in no significant time main effect, condition main effect, or time by condition interaction.

- Waist girth

The ANOVA with repeated measures on time resulted in a significant time main effect (F(1.7,139.2) = 6.47, *p* < 0.01, η_p_^2^ < 0.08), with waist girth decreasing from the baseline (98.62 ± 13.12) to week 12 (97.58 ± 13.32). This appears to be due to a significant decrease in waist girth measurement from the baseline to 12 weeks in both groups. Waist girth measures returned to baseline values at the three-month follow-up in both groups. There was no significant condition main effect or time by condition interaction.

#### 3.4.5. Quality of Life (QOL)

There was a significant time main effect, with no time by condition interaction effect, for: bodily pain (F(1.7,129.7) = 48.9, *p* < 0.01, η_p_^2^ < 0.39), with bodily pain increasing from the baseline (37.96 ± 18.62) to week 12 (62.46 ± 19.31) and the three-month follow-up (66.01 ± 19.84); general health (F(1.38,109.38) = 42.1, *p* < 0.01, η_p_^2^ < 0.35), with general health increasing from the baseline (52.04 ± 10.86) to week 12 (68.40 ± 16.02) and the three-month follow-up (68.33 ± 16.55); social functioning (F(2,158) = 23.3, *p* < 0.01, η_p_^2^ < 0.23), with social functioning increasing from the baseline (66.72 ± 16.46) to week 12 (79.77 ± 22.39) and the three-month follow-up (82.59 ± 19.11); and mental health (F(2,158) = 4.25, *p* < 0.05, η_p_^2^ < 0.05), with mental health increasing from the baseline (66.33 ± 7.43) to week 12 (71.26 ± 12.29) and the three-month follow-up (69.14 ± 14.80). There was a significant time by condition interaction for role emotional (F(2,156) = 3.07, *p* < 0.05, η_p_^2^ < 0.04), which appears to be due to a significant decrease in the intervention group from week 12 to the three-month follow-up and a significant increase in the control group from week 12 to the three-month follow-up.

## 4. Discussion

The current study aimed to test the effects of an interactive online walking promotion tool on health status among cancer survivors, and to compare these effects between metropolitan and rural participants. For all measured variables, there were no differences in responses by region, suggesting that the strategy has the potential to narrow the gap in health status that is evident between metropolitan and rural cancer survivors [14,15]. For all but two outcome variables (6MWT and role emotional), there were significant time effects at the three-month follow-up that did not differ between the intervention and control groups. For all of these variables, other than bodily pain, the observed changes between the baseline and follow-up were indicative of improved health. These observations largely mirror the changes in steps/day observed in the same study, reported elsewhere [32], whereby the intervention group increased steps/day more than the control group across the intervention and at the three-month follow-up. Control group improvements in physical activity intervention trials have been previously reported, perhaps attributable to the provision of feedback to participants at each time point and recruitment of already motivated participants who are in the planning or action stages of behaviour change [41]. Cancer survivors in particular may have the desire to ‘take control’ of their health, with previous research suggesting that this can motivate cancer patients to exercise [42].

### 4.1. QOL

There were significant time main effects for bodily pain, general health, social functioning, and mental health. For role emotional, there was a condition main effect and a time by condition interaction. Higher scores on the SF-36v2 represent better health and lower scores represent more disability. This means that the intervention group experienced more limitations due to emotional problems and the control group experienced fewer limitations due to emotional problems at week 12. As physical activity has been associated with an improved emotional state [43], and improvements in mental health, general health, and social functioning were observed in both groups in the current study, the increase in perceived limitations due to emotional problems in the intervention group during the intervention is difficult to explain. Speculatively, this may be due to the intervention group being dependent on contact through the STRIDE website and goal setting, which ceased after the 12-week intervention, and the wait list control group expecting to receive access to the STRIDE website after the three-month follow-up. Qualitative investigations of cancer survivors’ emotional experiences during exposure to STRIDE supported this notion. Several participants discussed the difficulties in maintaining their motivation to walk after the program had finished: “I’m better off with somebody organizing me and telling me what to do”; and “since the three months has finished there has been really nothing from you guys to us, so I’ve been in control of it and that hasn’t really worked like it did when the project was happening” [44].

The main effect of bodily pain, with bodily pain increasing from the baseline to the end of the intervention and follow-up, may be explained by a range of factors, including muscle strain due to the increased walking or weather (arthritis worse in cold weather). Increased bodily pain over time is contrary to findings of other physical activity interventions in cancer survivors that reported improvements in QOL post-intervention [45,46,47]. A comparison of results across studies is difficult because QOL has been assessed using a range of different measures, including the Profile of Mood States (POMS) [45], Functional Assessment of Cancer Therapy (FACT) [46], and the European Organization for Research and Treatment of Cancer Quality of Life Questionnaire (EORTC QOL) [47]. Different modes of exercise intensity and duration were also used.

### 4.2. Physical Fitness (6MWT)

Compared with the baseline, meters walked in the intervention group improved by 4.3% after program completion and by 6.6% at the three-month follow-up. The control group improved by 1.2% after program completion and 1.3% at the three-month follow-up. The changes in the intervention group are quite modest compared with other similar studies. One physical activity study among cancer survivors reported increases of 14% and 17.7% in 6MWT distance in the intervention group at 12 weeks and the three-month follow-up, respectively [47]. Similarly, a supervised exercise study among stem cell transplant patients found improvements of 12% and 14% in 6MWT distance after the intervention and at the follow-up, respectively [48]. These relatively large reported increases in distance walked may be because the interventions were supervised ambulatory physical exercise programs incorporating both endurance and resistive strength exercises, compared with guided but unsupervised physical activity in the current study.

Fitness scores in the present study remained high at the three-month follow-up in both groups, while step counts in both groups decreased during this period [32]. A possible explanation for reduced step counts but maintained fitness is that participants may have chosen to engage in non-ambulatory exercise modalities (e.g., swimming, aqua-aerobics, cycling, gardening), which are not captured by pedometers that are only sensitive to vertical displacement of the hip [49]. Although survivors in the current study were encouraged to record non-ambulatory physical activities in their pedometer diaries, several participants forgot to record this information and therefore these data were not included in the analysis. Future studies could more accurately record non-ambulatory activities and therefore derive a measure of the total ‘volume’ of physical activity [50]. Another explanation for maintained fitness levels is that chronic physiological adaptations to exercise can persist for a period of time after exercise stimulus has been withdrawn [51]. Furthermore, a learning effect may account for increased 6MWTdistance at the three-month follow-up because participants may have become familiar with the task and better able to ‘pace’ themselves through the test. Some participants may have been competitive and wanted to beat their previous score. However, previous literature has demonstrated a good test-retest reliability for the six-min walk test among chronic disease populations [52,53].

### 4.3. Blood Pressure

A MANOVA revealed no significant time by condition effects for systolic or diastolic blood pressure. ANOVAs revealed that there were time main effects for systolic and diastolic blood pressure, with both blood pressure variables decreasing from the baseline to post-intervention and decreasing further to the three-month follow-up. The intervention group decreased systolic blood pressure by approximately 7 mmHg and the control group by approximately 9 mmHg from the baseline to week 24. The intervention group decreased their diastolic blood pressure by approximately 5 mmHg and the control group by approximately 3 mmHg from the baseline to week 24. The finding of decreased systolic blood pressure is consistent with published meta-analyses of effects of physical activity on blood pressure [54,55]. Reducing systolic blood pressure by 2 mmHg is associated with a 10% reduction in stroke mortality and a 7% reduction in mortality from vascular causes in middle-aged populations [56].

There may be several reasons for the decrease in blood pressure over time. Firstly, it is well-established that regular physical activity lowers blood pressure [54]. The increased walking during the STRIDE program may have contributed in part to the observed reduction in blood pressure. Secondly, anxiety raises blood pressure, by as much as 30 mmHg [57]. Often referred to as ‘white coat’ hypertension, this is commonly seen in patients who are anxious about a new situation or having their blood pressure taken. This reaction varies widely from patient to patient and may be abolished altogether with familiarization with the technique and circumstances of blood pressure measurement. Participants in the current study may have been relatively anxious at the first measurement session, but had become more familiar and comfortable with the testing process and research personnel by the second and third measurement sessions.

### 4.4. BMI

This study found no significant change in BMI for the intervention or control groups across time, contrary to other pedometer-based studies. A systematic review of pedometers and physical activity found that pedometer users experienced a reduction in BMI [58], which was more marked in studies: of older participants; with a higher proportion of Caucasians; that incorporated step goals; and were conducted over a longer duration. Further analysis in the current study indicates that there was a main effect of weight, with weight decreasing from the baseline to end-of-intervention, and further at the three-month follow-up in both the intervention and control groups. There was also a main effect for waist girth, with waist girth decreasing from the baseline to end-of-intervention. In line with these observations, it could be expected that BMI would also decrease. The reason for no change in BMI in this study may be due to the fact that BMI is a less sensitive measure than weight [59].

### 4.5. Waist Girth

There was a main effect of waist girth, with waist girth measures decreasing from the baseline to end of intervention, but regressing back to baseline values at the three-month follow-up. Waist girth is strongly associated with cardiovascular disease risk [60]. These findings are of particular importance as the participants in this study who decreased their waist girth, likely also favorably modified their cardiovascular risk. It is difficult to compare these findings with other physical activity intervention trials because, although many studies measure waist girth, it is often BMI that is reported. A pilot study investigating anthropometric changes in a walking intervention found decreases in waist girth from the baseline to end-of intervention (week 8), and these improvements in waist girth persisted at the three-month follow-up [61]. However, this study was conducted among African American breast cancer survivors, limiting the comparison of results of the current study to other populations.

## 5. Conclusions

The results of this study provide further evidence that pedometer interventions can produce favorable outcomes among survivors of mixed cancer types. This is promising for survivors in rural areas, whose barriers to physical activity may include the lack of access to exercise facilities. Devices such as pedometers may be sufficient for improving health outcomes and quality of life in cancer survivors for whom motivation for lifestyle change is already high [42]. This means health care professionals who are counselling their patients about physical activity could offer simple, inexpensive, and ‘light touch’ options beyond traditional fitness center-based recommendations. However, the outcomes of online programs such as STRIDE may be different for patients with specific types of cancer, or who live in rural areas where access to ongoing, long-term support from health professionals is relatively less available. Given that the STRIDE intervention was designed to encourage autonomy and intrinsic motivation [20,62], and therefore a higher likelihood of long-term maintenance of active lifestyles [63], future research should extend the follow-up period to explore the impact of the website beyond that due to simply the provision of a pedometer.

## Figures and Tables

**Table 1:** Socio-demographic and health characteristics of participants.

Characteristics	Intervention (*n* = 46)		Control (*n* = 45)	
	*n*	(%)	*n*	(%)
Age, y				
Mean	65.2		66.1	
SD	9.3		9.4	
Range	29–78		44–86	
Sex				
Male	21	(45.7)	23	(51.1)
Female	25	(54.3)	22	(48.9)
Location				
Metropolitan	24	(52.2)	24	(53.3)
Rural	22	(47.8)	21	(46.7)
Marital status				
Married/living	37	(80.4)	35	(77.8)
with partner				
Not married	9	(19.6)	10	(22.2)
Race/ethnicity				
Caucasian	45	(97.8)	42	(93.3)
Asian	0	(0)	2	(4.4)
ATSI	1	(2.2)	1	(2.2)
Education				
<High school	0	(0)	2	(4.4)
High school or diploma	31	(67.4)	35	(77.8)
University	15	(32.6)	7	(15.6)
SES (SEIFA)				
Low	18	(39.1)	18	(40.0)
Middle	18	(39.1)	14	(31.1)
High	10	(21.7)	12	(26.7)
BMI, kg/m^2^				
Normal (<25)	6	(13.0)	9	(20.0)
Overweight (25–29)	15	(32.6)	16	(35.6)
Obese (30–39)	23	(50.0)	18	(40.0)
Morbidly obese (40+)	2	(4.3)	2	(4.4)
Smoking status				
Smoker	0	(0)	4	(8.9)
Ex-smoker	11	(23.9)	19	(42.2)
Non-smoker	35	(76.1)	22	(48.9)
Length of time using Internet, y				
First time	1	(2.2)	4	(8.9)
<6–12 months	0	(0)	6	(13.3)
1–3	6	(13.0)	1	(2.2)
4–6	5	(10.9)	5	(11.1)
≥7	34	(73.9)	29	(64.4)

Abbreviations: BMI, Body Mass Index; ATSI, Aboriginal or Torres Strait Islander; SEIFA, Socio-Economic Indexes for Areas; SES, Socio Economic Status; y, years. Missing values are present where percentages do not add up to 100 percent.

**Table 2:** Cancer specific characteristic and comorbidities of participants.

Characteristics	Intervention (*n* = 46)		Control (*n* = 45)	
	*n*	(%)	*n*	(%)
Cancer type				
Breast	21	(45.7)	17	(37.8)
Prostate	9	(19.6)	11	(24.4)
Colorectal	5	(10.9)	4	(8.9)
Head and neck	3	(6.5)	3	(6.7)
Gynaecologic *	1	(2.2)	2	(4.4)
Lung	1	(2.2)	0	(0)
Other	6	(13.0)	8	(17.8)
No. of cancers				
1	39	(84.8)	39	(86.7)
>1	7	(15.2)	6	(13.3)
Time since first cancer diagnosis, y				
<2	3	(6.5)	1	(2.2)
2–5	25	(54.3)	26	(57.8)
6–9	11	(23.9)	6	(13.3)
≥10	6	(13.0)	9	(20.0)
Comorbidity				
Diabetes II	4	(8.7)	3	(6.5)
Arthritis	5	(10.9)	5	(11.1)
Hypertension	12	(26.1)	13	(28.3)
High cholesterol	10	(21.8)	9	(20.0)

Abbreviations: y, years. Note: * Gynaecologic cancers include cervical, uterine, and ovarian cancer. Missing values are present where percentages do not add up to 100 percent.

**Table 3:** The Cronbach’s α for the eight domains of the SF-36v2 questionnaire.

Dimensions	Cronbach’s α
Physical functioning	0.865
Role physical	0.926
Bodily pain	0.724
General health	0.809
Vitality	0.805
Social functioning	0.607
Role emotional	0.913
Mental health	0.827

**Table 4:** Health variables for intervention and control groups at baseline, week 12, and follow-up.

Health Indicators	Intervention			Control		
	Baseline	Week 12	Week 24	Baseline	Week 12	Week 24
Physical fitness (6MWT) (m) ^a^**	530.4	553.9	565.3	515.2	521.3	528.9
	(66.8)	(72.4)	(81.3)	(88.7)	(101.9)	(102.6)
Systolic blood pressure [mmHg] ^a^**	138.7	134.4	131.83	139.7	133.2	130.6
	(15.5)	(16.8)	(15.5)	(20.2)	(19.3)	(16.6)
Diastolic blood pressure [mmHg] ^a^**	81.3	77.2	75.7	79.8	79.4	76.5
	(9.6)	(9.4)	(10.2)	(10.8)	(12.1)	(11.0)
Waist girth (cm) ^a^**	99.7	98.6	99.8	97.6	96.8	97.8
	(13.1)	(13.3)	(13.5)	(13.3)	(13.4)	(13.1)
BMI (kg/m^2^)	30.4	30.2	30.1	28.6	28.6	28.6
	(4.9)	(4.9)	(4.9)	(4.5)	(4.4)	(4.4)

Abbreviations: ^a^ main effect of time. ** = significant < 0.01.

**Table 5:** SF-36v2 subscales for intervention and control groups at baseline, week 12, and follow-up.

SF-36v2 Subscales	Intervention			Control		
	Baseline	Week 12	Week 24	Baseline	Week 12	Week 24
Physical functioning	79.5	80.8	80.9	74.5	74.3	75.1
	(15.8)	(16.3)	(17.5)	(20.5)	(20.6)	(17.5)
Role physical	71.1	72.9	72.9	71.0	68.4	75.1
	(26.3)	(19.0)	(22.3)	(25.3)	(21.2)	(19.4)
Bodily pain ^a^**	40.5	63.3	65.7	35.5	61.6	66.3
	(20.8)	(18.5)	(18.2)	(16.1)	(20.3)	(21.5)
General health ^a^**	53.2	69.5	72.7	50.9	67.4	68.3
	(11.9)	(16.5)	(19.7)	(9.8)	(15.7)	(16.6)
Vitality	58.7	57.1	55.6	61.1	55.2	55.7
	(10.0)	(15.9)	(16.0)	(9.3)	(13.8)	(12.8)
Social functioning ^a^**	71.5	83.4	83.9	62.3	76.3	81.4
	(15.4)	(20.5)	(16.9)	(16.3)	(23.8)	(21.1)
Role emotional ^bc^*	86.8	86.3	81.2	74.2	74.5	78.5
	(18.5)	(16.7)	(22.9)	(22.0)	(21.4)	(18.1)
Mental health ^a^*	68.5	70.8	67.8	64.3	71.7	70.3
	(8.1)	(13.1)	(15.6)	(6.1)	(11.6)	(14.1)

Abbreviations: ^a^ main effect of time, ^b^ time by condition interaction, ^c^ condition main effect; * = significant < 0.05, ** = significant < 0.01.
